# Vulvar Pyogenic Granuloma in Adult Female Population: A Case Report and Review of the Literature

**DOI:** 10.1155/2021/5525092

**Published:** 2021-08-03

**Authors:** Nastaran Mahmoudnejad, Alireza Zadmehr, Mohammad Hamidi Madani

**Affiliations:** ^1^Assistant Professor of Urology, Fellowship of Female Urology, Labbafinejad Hospital, Urology and Nephrology Research Center, Shahid Beheshti University of Medical Sciences (SBMU), Tehran, Iran; ^2^Resident of Urology, Labbafinejad Hospital, Urology and Nephrology Research Center, Shahid Beheshti University of Medical Science (SBMU), Tehran, Iran; ^3^Department of Urology, Labbafinejad Hospital, Shahid Beheshti University of Medical Science (SBMU), Tehran, Iran

## Abstract

Pyogenic granuloma (PG) is an uncommon lesion of unknown etiology. It may be formed following a minor injury. They result from a reactive or inflammatory process consisting of proliferating vascular channels, immature fibroblastic connective tissue, and scattered inflammatory cells rather than neoplastic process. Bleeding is the most common symptom of the lesion. They may be seen in all age groups, and there is no clear predominance of a gender. Vulvar PGs can be confused with other polypoid or sessile lesions of the genital site. There are only a few cases of female genital PGs reported in the literature. Herein, we describe the first case of vulvar (clitoral) PG in an Iranian patient and a brief review of the literature in this regard.

## 1. Introduction

Lobular capillary hemangioma or pyogenic granuloma (PG) is a common acquired benign vascular lesion of the skin and mucous membranes. [1–3] PG is one of the most common vascular tumors in all ages and may be formed following a minor injury. [3] This hypervascularized lesion grows rapidly in a couple of weeks or months and usually presents as an erythematous, pedunculated, exophytic, or sessile lesion, with a smooth or lobulated surface [1–4]. PGs may be seen at different areas including the head, neck, and extremities or in the oral mucosa cavity during pregnancy [3–6]. Vulvar PG is a rare finding, and to our best knowledge, only twelve cases in adult female population have been described in the literature [3, 4, 6–12]. Herein, we introduce the first vulvar PG in an Iranian female patient and a brief review of the literature in this regard.

## 2. Case Presentation

**Figure 1:**
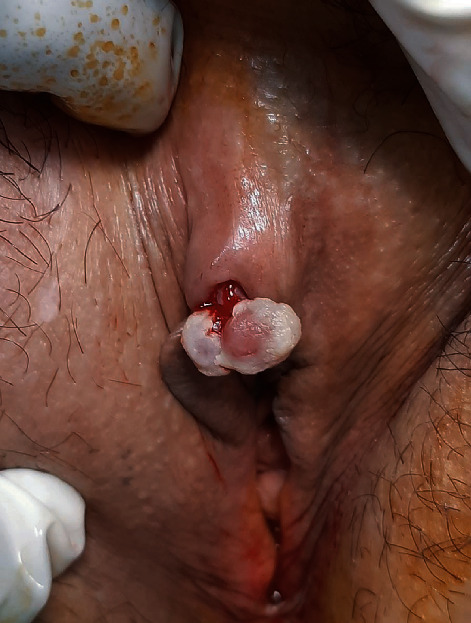
A 2.5 × 0.5 cm pedunculated and easy bleeding lesion beneath the clitoral hood.

**Figure 2:**
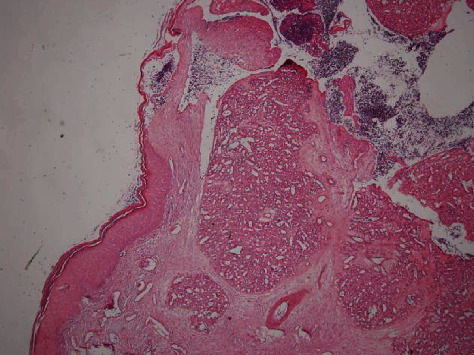
Ulcerated skin with a lobular growth pattern of capillary sized vessels (H&E, ×40).

A 54-year-old married woman complained of an easy bleeding, nontender, relatively fast growing clitoral lesion since 6 months ago. She did not mention any genital injury or trauma lately. In past medical history, we did not find anything contributory. Physical examination revealed a 2.5 × 0.5 cm, pedunculated, erythematous, granulation tissue-like appearance lesion beneath the clitoral hood (Figure 1). Vaginal examination was unremarkable, and routine lab tests were all within normal limits. She underwent surgical excision of the lesion under local anesthesia. We did not use any electrocautery in the region, and base of the mass was repaired with separate absorbable 3-0 sutures. She was discharged from the hospital at the same day. Postoperative course was uneventful. Histopathological evaluation revealed a 19.98 mm polypoid lesion with surface ulceration, composed of lobular configuration of capillaries within inflamed stroma compatible with PG (Figure 2). In the six-month postoperative visit, there was no recurrence or scar tissue at the surgical site. Tactile and sexual sensation of the clitoris was preserved.

## 3. Discussion

PG results from a reactive or inflammatory process, and it consists of proliferating vascular channels, immature fibroblastic connective tissue, and scattered inflammatory cells rather than neoplastic process. The exact etiology and pathogenesis of these lesions are not fully understood. However, it is assumed that minor trauma (in 50% of patients), chronic local irritation, hormonal influences, viral oncogenes, underlying microscopic arteriovenous malformation, and production of angiogenic factors may play a role in formation of them [5]. PG has variable variants including disseminated, subcutaneous, intravenous, and systemic medication-induced subtypes [9]. Bleeding is usually the leading symptom for a visit to the doctor's office. It can be profuse and refractory to conservative treatment [13].

These lesions may be seen in all age groups, and according to our literature review, there is no clear predominance of a gender [13]. Based on Arikan et al.'s study, there was a higher frequency of PG in the second decade of life and a female predilection of 2 : 1 [3]. A 10-year retrospective analysis of 82 cases by Akamatsu et al. [1] revealed a slight male gender preponderance in overall patients, and the head and neck areas, including the oral cavity and nasal mucosa, were the most affected sites in both sexes.

PGs are considered to be benign. However, vulvar PGs can cause confusion with some benign and malignant genital lesions including warts, giant condylomas, Kaposi's sarcoma, basal cell carcinoma, malignant melanoma, lymphomas, bowenoid papulosis, verrucous carcinoma, acquired tufted angioma, epithelioid hemangioendothelioma and bacillary angiomatosis [4, 13]. Approximately 63% of the patients may have an associated underlying systemic disorder like rheumatoid arthritis, hematological malignancies, and inflammatory bowel disease. In “localized genital” PGs, underlying systemic diseases are extremely rare [6]. Various cutaneous pathologies including port wine stains, psoriasis, eczema, burns, erythroderma, insect bites, and even cutaneous changes following retinoid therapy may be accompanied by PGs [9].

Invasive and minimally invasive treatments of PGs include surgical excision, curettage followed by electrocauterization, cryotherapy with liquid nitrogen, sclerotherapy, radiosurgery, silver nitrate cautery, microembolization, and lasers. Photodynamic therapy with 5-aminolevulinic acid has been used for single PG either [8, 13]. Several oral or topical treatments of PGs have been described in the literature. They include 5% imiquimod cream, timolol, propranolol, prednisolone, and clobetasol [5, 8, 11].

The current patient did not have an underlying cutaneous or systemic disease. She did not mention any trauma or minor surgeries at the vulvar region. Since the lesion was located just beneath the clitoral hood, we preferred not to use electrocautery in order to avoid probable thermal injury of clitoral neurovascular bundle. In the 6-month follow-up of the patient, tactile and sexual sensation of the clitoris was preserved and there was no recurrence at the surgical site. To our best knowledge, this is the first published report of vulvar PG in Iran. Due to the special role of the clitoris in female sexual function, we believe that in the management of the clitoral lesions, special care should be given to preserving dorsal clitoral nerve and associated vascular content. Meticulous dissection and minimizing electrocautery usage should be considered.

Complete surgical excision and primary closure are the most advised and acceptable treatment of vulvar PGs (Table 1). Since the definitive diagnosis of PG is made by histopathologic examination, a skin biopsy prior to any conservative or ablational therapy is recommended.

## 4. Conclusion

Although the vulva is a rare location of PG formation, it should be considered as a differential diagnosis of any polypoid or sessile lesion at this site. Since it sometimes may occur with underlying diseases or malignant conditions, taking medical history and performing physical examination are recommended. Complete surgical excision and primary closure of the lesion are the medical treatment of choice with very low recurrence rate. In clitoral PGs, in order to avoid compromising the neurovascular bundle of the clitoris, meticulous dissection and preventing usage of electrocauterization are highly suggested.

## Figures and Tables

**Table 1:** Reports of vulvar pyogenic granuloma in the literature.

Cases/first author	Year	Age	Description of lesions	Symptom/underlying disease	Treatment
(1) Somesh Gupta et al. [4]	2000	57	Multiple large pedunculated, papillomatous lesions, on the left labia major	Bleeding	Surgical excision
(2) Kian-Mei Chong et al. [11]	2005	52	Reddish, 3 × 2 mm, ulcerated papule on the right labia major	Bleeding	Surgical excision
(3) Deniz Cemgil Arikan et al. [3]	2011	57	Multiple large 2-4 cm, papillomatous, pedunculated, and lobulated lesions on the labia major and clitoris	Bleeding	Surgical excision
(4) Vani Malhotra et al. [9]	2012	52	A single 3 × 3 cm lesion with smooth surface on the left labia major	Pain	Surgical excision
(5) Masataka Satoh et al. [6]	2013	74	Granulomatous vulvar ulcer	Pain	Oral prednisolone
(6) F. Abreu-Dos-Santos et al. [12]	2016	51	Lobulated reddish 2 cm ulcerated lesion on the upper part of the right labia major	Bleeding	Surgical excision
(7) Mónica Vences Carranza et al. [7]	2017	55	Single exophytic hemispherical, erythematous 5 mm, pedunculated, smooth surface lesion on the left side of labia major	Bleeding	Surgical excision
(8) Mónica Vences Carranza et al. [7]	2017	54	Multiform erythematous 2.5 mm diameter, hard pedunculated lesion on the right side of labia major	—	Surgical excision
(9) Mónica Vences Carranza et al. [7]	2017	—	Multiple exophytic erythematous 0.5 cm in diameter, pedunculated lesions of the vulva and clitoral hood	Previous history of lichen sclerosis	Surgical excision
(10) Shivani Ranjan and BD Thakur [8]	2018	23	Solitary cherry red pedunculated lesion on the lower aspect of the left labia major	Pain and bleeding	Surgical excision
(11) Shivani Ranjan and BD Thakur [8]	2018	48	A single, erythematous, pedunculated lesion with lobulated surface on the left labia major	Bleeding	Surgical excision
(12) Carlos Cuenca-Barrales et al. [10]	2018	18	Multiple ulcerated exophytic dome-shaped lesions in the intergluteal fold and pubic region	Previous history of acne	Reduction of isotretinoin dosage and topical application of clobetasol
(13) Present case	2020	54	Solitary pedunculated, granulation tissue-like lesion, beneath the clitoral hood	Spotting	Surgical excision
